# Crystal structure of 4,4′-(ethene-1,2-di­yl)dipyridinium bis­(3-carb­oxy­benzene­sulfonate)

**DOI:** 10.1107/S1600536814022673

**Published:** 2014-10-18

**Authors:** Jing Wu, Long-Guan Zhu

**Affiliations:** a Department of Chemistry, Zhejiang University, Hangzhou, Zhejiang 310027, People’s Republic of China

**Keywords:** crystal structure, 3-sulfobenzoate, 1,2-bis­(pyridin-4-yl)ethyl­ene, hydrogen bonding, 4,4′-(ethane-1,2-di­yl)dipyridinium

## Abstract

In the title mol­ecular salt, C_12_H_12_N_2_^2+^·2C_7_H_5_O_5_S^−^, the complete dication is generated by crystallographic inversion symmetry. In the anion, the sulfonic acid group is deprotonated and the dihedral angle between the planes of the carb­oxy­lic acid group and the benzene ring is 12.41 (11)°. In the crystal, the anions are linked into inversion dimers by pairs of O—H⋯O hydrogen bonds, which generate *R*_2_^2^(16) loops. The dications link the anion dimers into [10-2] chains *via* N—H⋯O hydrogen bonds.

## Related literature   

For general background to salts of 1,2-bis­(pyridin-4-yl)ethyl­ene and sulfobenzoates and their applications, see: Ma & Zhu (2014 ▶); Zheng & Zhu (2014 ▶); Lesniewska *et al.* (2014 ▶); Danylyuk *et al.* (2010 ▶); Zhang & Zhu (2006 ▶, 2007 ▶).
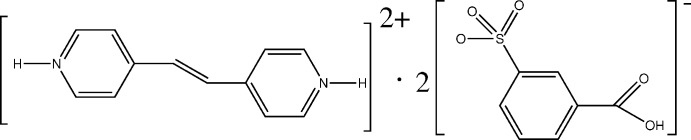


## Experimental   

### Crystal data  

C_12_H_12_N_2_^2+^·2C_7_H_5_O_5_S^−^
*M* *_r_* = 586.58
Triclinic, 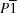

*a* = 7.4573 (5) Å
*b* = 7.8381 (6) Å
*c* = 11.3111 (9) Å
α = 85.525 (6)°
β = 86.634 (6)°
γ = 69.545 (7)°
*V* = 617.22 (8) Å^3^
*Z* = 1
Mo *K*α radiation
μ = 0.28 mm^−1^
*T* = 170 K
0.43 × 0.29 × 0.18 mm

### Data collection  

Oxford Diffraction Xcalibur (Atlas, Gemini Ultra CCD) diffractometer
Absorption correction: multi-scan (*CrysAlis PRO*; Oxford Diffraction, 2009 ▶) *T*_min_ = 0.888, *T*_max_ = 0.951
3834 measured reflections
2182 independent reflections
1899 reflections with *I* > 2σ(*I*)
*R*_int_ = 0.025

### Refinement  

*R*[*F* ^2^ > 2σ(*F* ^2^)] = 0.036
*wR*(*F* ^2^) = 0.096
*S* = 0.98
2182 reflections
187 parameters
2 restraints
H atoms treated by a mixture of independent and constrained refinement
Δρ_max_ = 0.24 e Å^−3^
Δρ_min_ = −0.42 e Å^−3^

### 

Data collection: *CrysAlis PRO* (Oxford Diffraction, 2009 ▶); cell refinement: *CrysAlis PRO*; data reduction: *CrysAlis PRO*; program(s) used to solve structure: *SHELXS97* (Sheldrick, 2008 ▶); program(s) used to refine structure: *SHELXL97* (Sheldrick, 2008 ▶); molecular graphics: *ORTEP-3 for Windows* (Farrugia, 2012 ▶); software used to prepare material for publication: *WinGX* (Farrugia, 2012 ▶).

## Supplementary Material

Crystal structure: contains datablock(s) I, global. DOI: 10.1107/S1600536814022673/hb7296sup1.cif

Structure factors: contains datablock(s) I. DOI: 10.1107/S1600536814022673/hb7296Isup2.hkl

Supporting information file. DOI: 10.1107/S1600536814022673/hb7296Isup3.cml

**Fig. 1.**
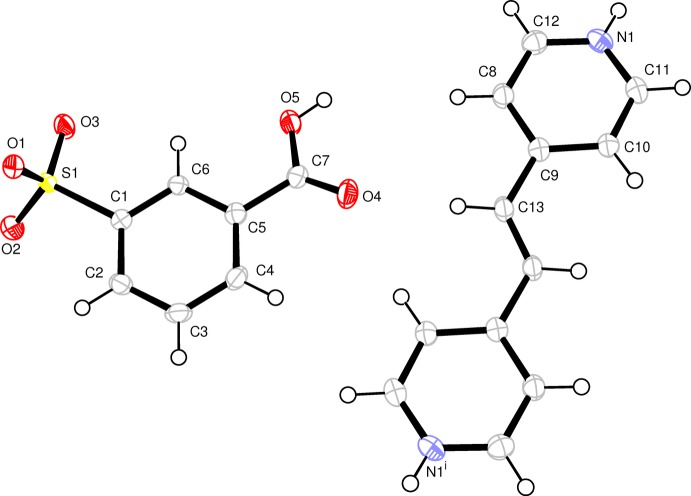
View of the asymmetry unit of (I) showing displacement ellipsoids at the 50% probability level. Symmetry code: (i) 2–x, –y, 1–z.

. DOI: 10.1107/S1600536814022673/hb7296fig1.tif
View of the asymmetry unit of (I) showing displacement ellipsoids at the 50% probability level. Symmetry code: (i) 2–x, –y, 1–z.

**Fig. 2.**
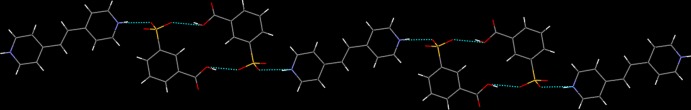
The hydrogen-bonded chain of (I).

. DOI: 10.1107/S1600536814022673/hb7296fig2.tif
The hydrogen-bonded chain of (I).

CCDC reference: 1029402

Additional supporting information:  crystallographic information; 3D view; checkCIF report

## Figures and Tables

**Table 1:** Hydrogen-bond geometry (, )

*D*H*A*	*D*H	H*A*	*D* *A*	*D*H*A*
O5H5*A*O3^i^	0.86(1)	1.85(1)	2.683(2)	163(3)
N1H1*A*O1^ii^	0.83(1)	1.91(1)	2.727(2)	172(4)

Symmetry codes: (i) 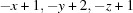; (ii) 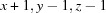.

## supplementary crystallographic information

### S1. Comment

Sulfobenzoate derivatives and their organic or coordination compounds are very interesting in material science, such as potential applications in fluorescence and catalytic fields (Ma & Zhu, 2014; Zheng & Zhu, 2014). Sulfobenzoates have two functional groups, sulfonate and carboxylate, and can coordinate to metal ions *via* versatile coordination modes or can form abundant hydrogen bonds due to they have five donors or acceptors. The 1,2-bis(pyridin-4-yl)ethylene derivatives have been widely used as bridge linkers in the coordination chemistry. several organic complexes with the 1,2-bis(pyridin-4-yl)ethylene and sulfobenzoate ligands have been reported, such as, 4,4'-ethylene-1,2-diyldipyridinium bis(4-carboxybenzenesulfonate) dihydrate (Zheng & Zhu, 2014), bis(4-(2-(pyridin-4-yl)vinyl)pyridinium) 4-sulfonatobenzoate trihydrate (Zhang & Zhu, 2006), and 4-(2-(pyridin-4-yl)ethenyl)pyridinium 2-carboxybenzenesulfonate (Zhang & Zhu, 2007). The title complound has 1:2 ratio of cation to anion without any water molecule (Fig. 1). The cation is protonated and the anion is partly deprotonated. Two pyridyl rings of the 4-(2-(pyridin-4-yl)ethenyl)pyridinium anion are coplanar and the whole cation is a big pi-conjugated system. Two anions are linked by O—H···O between sulfonate and carboxylate groups into a dimer and these anionic dimers interact with cations by N—H···O hydrogen bonds, generating a chain (Fig. 2).

### S2. Experimental

A mixed solution of 3-sulfobenzoate sodium (0.224 g,1 mmol) in 10 ml CH_3_OH and 1,2-bis(pyridin-4-yl)ethylene(0.091 g, 0.5 mmol) in 8 ml of CH_3_CN was stirred for one hour and set aside for slow evaporation at room tempereture. After two days, yellow plates were obtained and collected by filtration.

### S3. Refinement

The nitrogen and carboxylate H atoms were found in the Fourier map with fixed *U*_iso_=0.08 Å^2^. The other H atoms were positioned geometrically and allowed to ride on their parent atoms at distances of C—H=0.93 Å (for C*sp*^2^) with *U*_iso_=1.2*U*_eq_(parent atom).

### Crystal data

**Table:**

C_12_H_12_N_2_^2+^·2C_7_H_5_O_5_S^−^	*Z* = 1
*M**_r_* = 586.58	*F*(000) = 304
Triclinic, *P*1	*D*_x_ = 1.578 Mg m^−^^3^
Hall symbol: -P 1	Mo *K*α radiation, λ = 0.71073 Å
*a* = 7.4573 (5) Å	Cell parameters from 1621 reflections
*b* = 7.8381 (6) Å	θ = 3.3–29.4°
*c* = 11.3111 (9) Å	µ = 0.28 mm^−^^1^
α = 85.525 (6)°	*T* = 170 K
β = 86.634 (6)°	Plate, yellow
γ = 69.545 (7)°	0.43 × 0.29 × 0.18 mm
*V* = 617.22 (8) Å^3^	

### Data collection

**Table:**

Oxford Diffraction Xcalibur (Atlas, Gemini Ultra CCD) diffractometer	2182 independent reflections
Radiation source: fine-focus sealed tube	1899 reflections with *I* > 2σ(*I*)
Graphite monochromator	*R*_int_ = 0.025
ω scans	θ_max_ = 25.1°, θ_min_ = 3.3°
Absorption correction: multi-scan (*CrysAlis PRO*; Oxford Diffraction, 2009)	*h* = −8→8
*T*_min_ = 0.888, *T*_max_ = 0.951	*k* = −8→9
3834 measured reflections	*l* = −13→11

### Refinement

**Table:**

Refinement on *F*^2^	Primary atom site location: structure-invariant direct methods
Least-squares matrix: full	Secondary atom site location: difference Fourier map
*R*[*F*^2^ > 2σ(*F*^2^)] = 0.036	Hydrogen site location: inferred from neighbouring sites
*wR*(*F*^2^) = 0.096	H atoms treated by a mixture of independent and constrained refinement
*S* = 0.98	*w* = 1/[σ^2^(*F*_o_^2^) + (0.0444*P*)^2^ + 0.4291*P*] where *P* = (*F*_o_^2^ + 2*F*_c_^2^)/3
2182 reflections	(Δ/σ)_max_ = 0.001
187 parameters	Δρ_max_ = 0.24 e Å^−^^3^
2 restraints	Δρ_min_ = −0.42 e Å^−^^3^

### Special details

**Table:**

Experimental. Absorption correction: CrysAlisPro, Oxford Diffraction Ltd., Version 1.171.33.53 (release 17-11-2009 CrysAlis171 .NET) (compiled Nov 17 2009,16:58:22) Empirical absorption correction using spherical harmonics, implemented in SCALE3 ABSPACK scaling algorithm.
Geometry. All e.s.d.'s (except the e.s.d. in the dihedral angle between two l.s. planes) are estimated using the full covariance matrix. The cell e.s.d.'s are taken into account individually in the estimation of e.s.d.'s in distances, angles and torsion angles; correlations between e.s.d.'s in cell parameters are only used when they are defined by crystal symmetry. An approximate (isotropic) treatment of cell e.s.d.'s is used for estimating e.s.d.'s involving l.s. planes.
Refinement. Refinement of *F*^2^ against ALL reflections. The weighted *R*-factor *wR* and goodness of fit *S* are based on *F*^2^, conventional *R*-factors *R* are based on *F*, with *F* set to zero for negative *F*^2^. The threshold expression of *F*^2^ > σ(*F*^2^) is used only for calculating *R*-factors(gt) *etc*. and is not relevant to the choice of reflections for refinement. *R*-factors based on *F*^2^ are statistically about twice as large as those based on *F*, and *R*- factors based on ALL data will be even larger.

### Fractional atomic coordinates and isotropic or equivalent isotropic displacement parameters (Å^2^)

**Table:**

	*x*	*y*	*z*	*U*_iso_*/*U*_eq_	
S1	0.19563 (7)	1.18509 (7)	0.82396 (4)	0.02058 (17)	
N1	1.2183 (2)	0.1796 (3)	0.13817 (15)	0.0249 (4)	
H1A	1.254 (5)	0.198 (5)	0.0693 (13)	0.080*	
O1	0.3295 (2)	1.2088 (2)	0.90567 (12)	0.0262 (3)	
O2	0.0064 (2)	1.2254 (2)	0.87794 (13)	0.0297 (4)	
O3	0.1989 (2)	1.2820 (2)	0.70976 (13)	0.0309 (4)	
O4	0.6933 (2)	0.4749 (2)	0.56362 (13)	0.0313 (4)	
O5	0.5777 (2)	0.7626 (2)	0.48846 (14)	0.0339 (4)	
H5A	0.656 (4)	0.725 (5)	0.430 (2)	0.080*	
C1	0.2852 (3)	0.9481 (3)	0.79837 (17)	0.0197 (4)	
C2	0.2567 (3)	0.8244 (3)	0.88595 (18)	0.0242 (5)	
H2	0.1840	0.8660	0.9543	0.029*	
C3	0.3371 (3)	0.6395 (3)	0.87079 (19)	0.0271 (5)	
H3	0.3179	0.5567	0.9291	0.032*	
C4	0.4461 (3)	0.5766 (3)	0.76930 (19)	0.0258 (5)	
H4	0.5019	0.4519	0.7603	0.031*	
C5	0.4716 (3)	0.7008 (3)	0.68091 (17)	0.0206 (4)	
C6	0.3908 (3)	0.8874 (3)	0.69538 (17)	0.0196 (4)	
H6	0.4076	0.9706	0.6364	0.024*	
C7	0.5925 (3)	0.6311 (3)	0.57261 (18)	0.0231 (5)	
C8	1.0500 (3)	0.2889 (3)	0.31413 (18)	0.0246 (5)	
H8	0.9812	0.3866	0.3592	0.029*	
C9	1.0830 (3)	0.1109 (3)	0.36022 (17)	0.0204 (4)	
C10	1.1909 (3)	−0.0318 (3)	0.29100 (18)	0.0233 (5)	
H10	1.2187	−0.1522	0.3200	0.028*	
C11	1.2553 (3)	0.0070 (3)	0.18050 (18)	0.0255 (5)	
H11	1.3260	−0.0879	0.1338	0.031*	
C12	1.1187 (3)	0.3205 (3)	0.20264 (19)	0.0269 (5)	
H12	1.0961	0.4394	0.1719	0.032*	
C13	1.0020 (3)	0.0799 (3)	0.47750 (18)	0.0228 (4)	
H13	0.9494	0.1797	0.5238	0.027*	

### Atomic displacement parameters (Å^2^)

**Table:**

	*U*^11^	*U*^22^	*U*^33^	*U*^12^	*U*^13^	*U*^23^
S1	0.0253 (3)	0.0180 (3)	0.0175 (3)	−0.0069 (2)	0.00549 (19)	−0.0025 (2)
N1	0.0229 (9)	0.0334 (11)	0.0187 (9)	−0.0105 (8)	0.0001 (7)	−0.0006 (8)
O1	0.0315 (8)	0.0256 (8)	0.0249 (8)	−0.0138 (6)	0.0056 (6)	−0.0080 (6)
O2	0.0277 (8)	0.0274 (9)	0.0327 (9)	−0.0085 (7)	0.0088 (6)	−0.0058 (7)
O3	0.0460 (9)	0.0195 (8)	0.0206 (8)	−0.0048 (7)	0.0085 (6)	−0.0004 (6)
O4	0.0364 (9)	0.0205 (8)	0.0320 (9)	−0.0030 (7)	0.0022 (6)	−0.0070 (7)
O5	0.0473 (10)	0.0223 (8)	0.0260 (9)	−0.0066 (7)	0.0134 (7)	−0.0033 (7)
C1	0.0201 (10)	0.0190 (10)	0.0202 (10)	−0.0071 (8)	−0.0011 (7)	−0.0009 (8)
C2	0.0275 (11)	0.0283 (12)	0.0199 (11)	−0.0139 (9)	0.0025 (8)	−0.0023 (9)
C3	0.0338 (12)	0.0255 (12)	0.0248 (11)	−0.0153 (9)	0.0001 (9)	0.0047 (9)
C4	0.0311 (12)	0.0181 (11)	0.0291 (12)	−0.0094 (9)	−0.0012 (9)	−0.0030 (9)
C5	0.0199 (10)	0.0221 (11)	0.0211 (11)	−0.0084 (8)	−0.0025 (8)	−0.0025 (8)
C6	0.0224 (10)	0.0199 (11)	0.0180 (10)	−0.0091 (8)	0.0004 (8)	−0.0008 (8)
C7	0.0259 (11)	0.0218 (11)	0.0232 (11)	−0.0098 (9)	−0.0007 (8)	−0.0042 (9)
C8	0.0271 (11)	0.0228 (11)	0.0222 (11)	−0.0063 (9)	0.0012 (8)	−0.0042 (9)
C9	0.0177 (10)	0.0222 (11)	0.0205 (10)	−0.0056 (8)	−0.0024 (7)	−0.0029 (8)
C10	0.0233 (11)	0.0215 (11)	0.0235 (11)	−0.0059 (8)	0.0016 (8)	−0.0027 (9)
C11	0.0226 (11)	0.0289 (12)	0.0238 (11)	−0.0067 (9)	0.0013 (8)	−0.0074 (9)
C12	0.0269 (11)	0.0253 (12)	0.0282 (12)	−0.0089 (9)	−0.0030 (9)	0.0015 (9)
C13	0.0240 (11)	0.0213 (10)	0.0212 (11)	−0.0048 (8)	0.0025 (8)	−0.0060 (8)

### Geometric parameters (Å, º)

**Table:**

S1—O2	1.4426 (15)	C4—C5	1.392 (3)
S1—O3	1.4499 (15)	C4—H4	0.9300
S1—O1	1.4664 (15)	C5—C6	1.393 (3)
S1—C1	1.781 (2)	C5—C7	1.497 (3)
N1—C11	1.338 (3)	C6—H6	0.9300
N1—C12	1.339 (3)	C8—C12	1.370 (3)
N1—H1A	0.827 (10)	C8—C9	1.392 (3)
O4—C7	1.200 (3)	C8—H8	0.9300
O5—C7	1.327 (3)	C9—C10	1.395 (3)
O5—H5A	0.855 (10)	C9—C13	1.464 (3)
C1—C6	1.386 (3)	C10—C11	1.363 (3)
C1—C2	1.392 (3)	C10—H10	0.9300
C2—C3	1.381 (3)	C11—H11	0.9300
C2—H2	0.9300	C12—H12	0.9300
C3—C4	1.386 (3)	C13—C13^i^	1.324 (4)
C3—H3	0.9300	C13—H13	0.9300
			
O2—S1—O3	113.77 (9)	C1—C6—C5	119.45 (18)
O2—S1—O1	111.53 (9)	C1—C6—H6	120.3
O3—S1—O1	112.10 (9)	C5—C6—H6	120.3
O2—S1—C1	107.31 (9)	O4—C7—O5	123.92 (19)
O3—S1—C1	106.65 (9)	O4—C7—C5	123.93 (19)
O1—S1—C1	104.84 (9)	O5—C7—C5	112.14 (17)
C11—N1—C12	121.56 (18)	C12—C8—C9	120.2 (2)
C11—N1—H1A	118 (3)	C12—C8—H8	119.9
C12—N1—H1A	120 (3)	C9—C8—H8	119.9
C7—O5—H5A	112 (2)	C8—C9—C10	118.08 (18)
C6—C1—C2	120.52 (19)	C8—C9—C13	119.46 (18)
C6—C1—S1	120.31 (15)	C10—C9—C13	122.45 (19)
C2—C1—S1	119.08 (15)	C11—C10—C9	119.4 (2)
C3—C2—C1	119.64 (19)	C11—C10—H10	120.3
C3—C2—H2	120.2	C9—C10—H10	120.3
C1—C2—H2	120.2	N1—C11—C10	121.0 (2)
C2—C3—C4	120.50 (19)	N1—C11—H11	119.5
C2—C3—H3	119.7	C10—C11—H11	119.5
C4—C3—H3	119.7	N1—C12—C8	119.7 (2)
C3—C4—C5	119.72 (19)	N1—C12—H12	120.1
C3—C4—H4	120.1	C8—C12—H12	120.1
C5—C4—H4	120.1	C13^i^—C13—C9	124.8 (2)
C4—C5—C6	120.14 (18)	C13^i^—C13—H13	117.6
C4—C5—C7	119.23 (18)	C9—C13—H13	117.6
C6—C5—C7	120.59 (18)		
			
O2—S1—C1—C6	140.83 (16)	C7—C5—C6—C1	−177.69 (17)
O3—S1—C1—C6	18.56 (18)	C4—C5—C7—O4	−11.6 (3)
O1—S1—C1—C6	−100.47 (16)	C6—C5—C7—O4	166.22 (19)
O2—S1—C1—C2	−42.65 (18)	C4—C5—C7—O5	169.47 (18)
O3—S1—C1—C2	−164.92 (15)	C6—C5—C7—O5	−12.7 (3)
O1—S1—C1—C2	76.05 (17)	C12—C8—C9—C10	1.6 (3)
C6—C1—C2—C3	1.1 (3)	C12—C8—C9—C13	−177.52 (19)
S1—C1—C2—C3	−175.44 (15)	C8—C9—C10—C11	−1.8 (3)
C1—C2—C3—C4	0.2 (3)	C13—C9—C10—C11	177.25 (18)
C2—C3—C4—C5	−1.3 (3)	C12—N1—C11—C10	0.6 (3)
C3—C4—C5—C6	1.1 (3)	C9—C10—C11—N1	0.8 (3)
C3—C4—C5—C7	178.94 (18)	C11—N1—C12—C8	−0.9 (3)
C2—C1—C6—C5	−1.2 (3)	C9—C8—C12—N1	−0.2 (3)
S1—C1—C6—C5	175.28 (14)	C8—C9—C13—C13^i^	169.0 (2)
C4—C5—C6—C1	0.1 (3)	C10—C9—C13—C13^i^	−10.1 (4)

Symmetry code: (i) −*x*+2, −*y*, −*z*+1.

### Hydrogen-bond geometry (Å, º)

**Table:**

*D*—H···*A*	*D*—H	H···*A*	*D*···*A*	*D*—H···*A*
O5—H5*A*···O3^ii^	0.86 (1)	1.85 (1)	2.683 (2)	163 (3)
N1—H1*A*···O1^iii^	0.83 (1)	1.91 (1)	2.727 (2)	172 (4)

Symmetry codes: (ii) −*x*+1, −*y*+2, −*z*+1; (iii) *x*+1, *y*−1, *z*−1.
