# Supplementary figures and images for: Crystal structure of 4,4′-(ethene-1,2-di­yl)dipyridinium bis­(3-carb­oxy­benzene­sulfonate)
Source: Acta Crystallogr Sect E Struct Rep Online. 2014 Oct 18;70(Pt 11):o1170. doi: 10.1107/S1600536814022673 (PMC4257284; doi:10.1107/S1600536814022673)

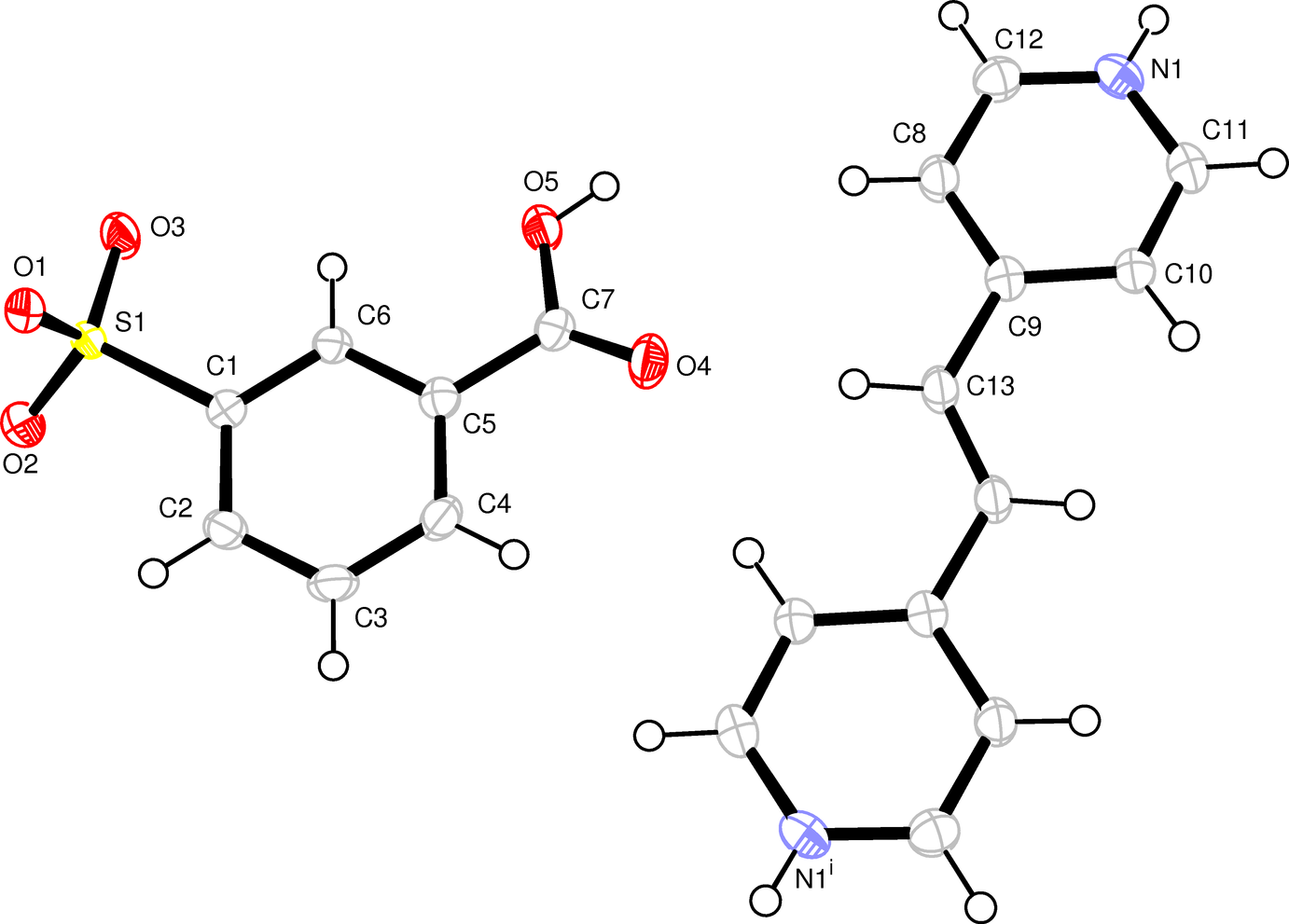

Supplement: Supplementary file 4 [file e-70-o1170-fig1.tif]

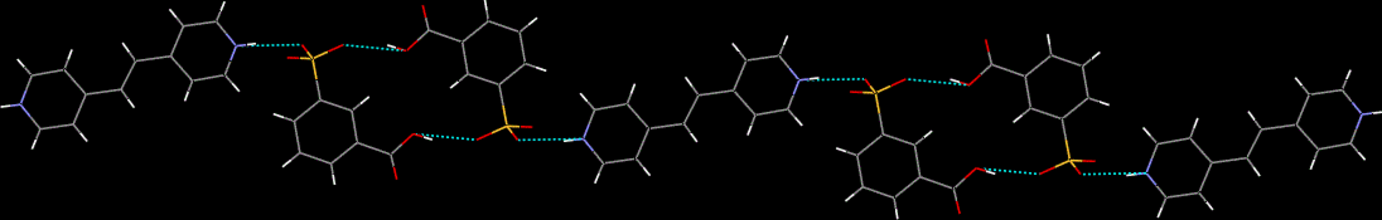

Supplement: Supplementary file 5 [file e-70-o1170-fig2.tif]
